# Effect of Red and Grey Selenium Nanoparticles on Yeast Growth: Short Communication

**DOI:** 10.3390/foods14244229

**Published:** 2025-12-09

**Authors:** Aya Ferroudj, Dávid Semsey, Daniella Sári, József Prokisch

**Affiliations:** 1 Nanofood Laboratory, Department of Animal Husbandry, Faculty of Agricultural and Food Sciences and Environmental Management, Institute of Animal Science, Biotechnology and Nature Conservation, University of Debrecen, 138 Böszörményi Street, 4032 Debrecen, Hungary; ferroudj.aya@agr.unideb.hu (A.F.); semi@gmail.hu (D.S.); saridaniella91@gmail.com (D.S.); 2 Doctoral School of Animal Husbandry, University of Debrecen, Böszörményi Street 138, 4032 Debrecen, Hungary; 3 Doctoral School of Nutrition and Food Science, University of Debrecen, 4032 Debrecen, Hungary

**Keywords:** selenium, nanoparticles, yeast, amorphous, crystallin, growth rate, nanoparticle toxicity, foam

## Abstract

Selenium nanoparticles (SeNPs) present a promising alternative to toxic inorganic selenium salts, yet the differential bioactivity between their allotropic forms—amorphous red (RSeNPs) and crystalline grey (GSeNPs)—is not fully determined. This study investigated the allotropic status and concentration-dependent effects of RSeNPs and GSeNPs (0.5, 5, and 50 mg·L^−1^) on *Saccharomyces cerevisiae* growth, monitored via foam expansion distance, calculated growth rate, and the normal logarithm of the samples’ optical densities at 600 nm. The results revealed that the allotropic form was the dominant factor influencing yeast performance. Specifically, RSeNPs exhibited superior biocompatibility; the 0.5 mg·L^−1^ dose (RSe0.5) yielded the highest overall growth rate, suggesting a potential growth-promoting effect. Conversely, GSeNPs demonstrated concentration-dependent toxicity, with the 50 mg·L^−1^ dose (GSe50) causing a statistically significant inhibition compared to the control. Moreover, optical density measurements confirmed that both red and grey SeNPs enhanced the maximum specific growth rate (µmax) compared to the control, demonstrating a stimulatory effect on yeast growth kinetics. These findings confirm that amorphous RSeNPs are less inhibitory and potentially more beneficial than their crystalline grey counterparts, underscoring the critical importance of nanoparticle morphology in determining biological outcomes.

## 1. Introduction

Selenium (Se) is a vital micronutrient essential for numerous biological processes, playing key roles in antioxidant defence, thyroid hormone metabolism, and immune function in humans and animals [1]. While selenium is crucial, its conventional inorganic forms (like selenite and selenate) often exhibit a narrow therapeutic window due to their inherent toxicity at higher concentrations [2]. Regarding the role and relevance of selenium nanoparticles, the development of selenium nanoparticles (SeNPs) represents a promising strategy to overcome the toxicity and bioavailability challenges associated with traditional selenium supplements. In this nanoscale form, selenium displays unique physicochemical properties, including enhanced bioavailability and significantly reduced toxicity compared to its inorganic salts [3]. A critical feature of SeNPs is their ability to exist in different allotropic forms, such as the amorphous red SeNPs and the crystalline, more stable grey (trigonal) SeNPs [4,5]. These forms are known to differ markedly in their stability, reactivity, and potential biological interactions [5,6]. However, comparative studies directly evaluating the differential bioactivity of red versus grey SeNPs remain limited. To select model organisms and systematically investigate the biological effects of these two distinct SeNPs forms, we utilize yeast (*Saccharomyces cerevisiae*) as a convenient and well-established eukaryotic model organism. Yeast offers several experimental advantages, including simple growth requirements, rapid replication kinetics, and high sensitivity to various environmental and chemical stressors; additionally, it plays a central role in food fermentation, including bread, beer, and wine production. Understanding how selenium nanoparticles affect yeast viability and fermentation activity is important both for food safety and for the development of nano-enabled functional ingredients [7,8]. Assessing the effect of SeNPs on yeast growth provides a cost-effective and rapid approach to screen for the potential cytotoxic or growth-promoting properties of these nanomaterials and has direct relevance for food biotechnology and nano-fortification strategies. This study aimed to synthesize red and grey SeNPs and comparatively evaluate the impact of these two allotropic forms on *Saccharomyces cerevisiae* growth across a range of concentrations.

## 2. Materials and Methods

### 2.1. Synthesis and Characterization of Selenium Nanoparticles

The production method is described in [4]. Red SeNPs were synthesized via chemical reduction. Sodium selenite (0.345 g) was accurately weighed and dissolved in 100 mL of distilled water. Similarly, ascorbic acid (0.352 g) was dissolved in 100 mL of distilled water. The reaction mixture was prepared by combining 1 mL of the sodium selenite solution with 9 mL of the ascorbic acid solution. The immediate formation of a red colloidal suspension indicated the successful synthesis of Red SeNPs, resulting in a stock solution with a selenium (Se) concentration of 158 mg·L^−1^. Grey SeNPs were subsequently obtained by subjecting the prepared Red SeNP colloidal suspension to heat treatment at 80 °C for 10 min. This process induced a phase transformation, yielding the grey SeNP form. SeNPs synthesized via this protocol typically exhibit particle sizes ranging between 80 and 120 nm for red SeNPs, while grey SeNPs show a slightly broader distribution of 90–150 nm based on DLS and TEM. UV-Vis spectrophotometry confirmed the purity of both nanoparticle types to be above 95%, with no detectable residual selenium species such as selenite or selenate. These physicochemical properties were validated in our laboratory using the same synthesis protocol.

### 2.2. Preparation of Yeast Growth Medium

The yeast growth medium was prepared by dissolving 2 g of sugar and 0.5 g of dry yeast in 100 mL of warm distilled water. The mixture was then allowed to activate for 10–15 min at room temperature prior to use in the experiments.

#### Experimental Design

The experiment was conducted in tubes, each containing a final reaction volume of 10 mL. The experimental treatments were designed to test four different final selenium concentrations (mg·L^−1^ Se) across both the red and grey SeNP types:

- Control (0 mg·L^−1^ Se);
- Low dose: 0.5 mg·L^−1^ SeNPs;
- Medium dose: 5 mg·L^−1^ SeNPs;
- High dose: 50 mg·L^−1^ SeNP.

Each treatment level was applied to both the red and grey SeNP suspensions, with three independent replicates per group (for a total of 3 treatments × 2 SeNP types + control) × 3 replicates = 21 tubes.

The required volumes of the 158 mg·L^−1^ SeNP stock solution needed to achieve the target concentrations in the final 10 mL volume were calculated as follows:
**Target Se Concentration (mg·L^−1^)****SeNP Stock Volume (μL)****Yeast Medium Volume (mL)****Total Volume (mL)**0.5329.9681053169.684105031656.835100 control01010

### 2.3. Incubation and Growth Monitoring

**Figure 1:**
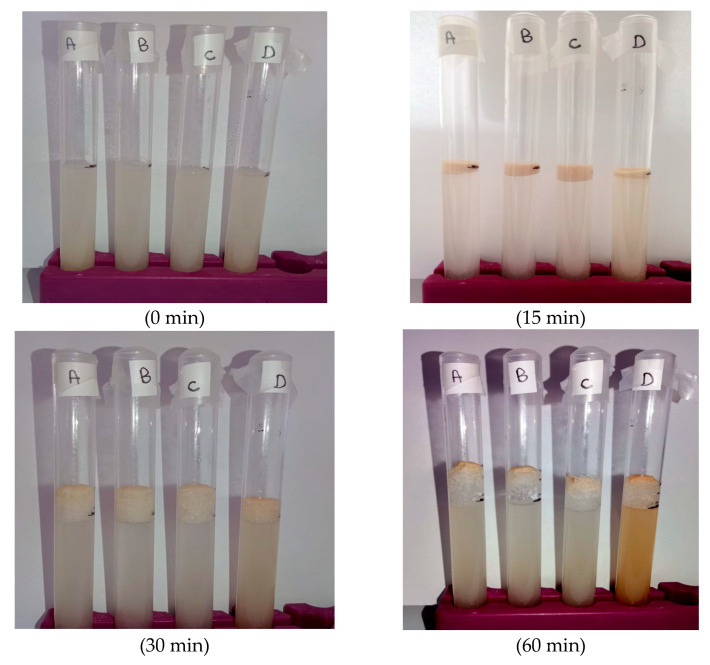

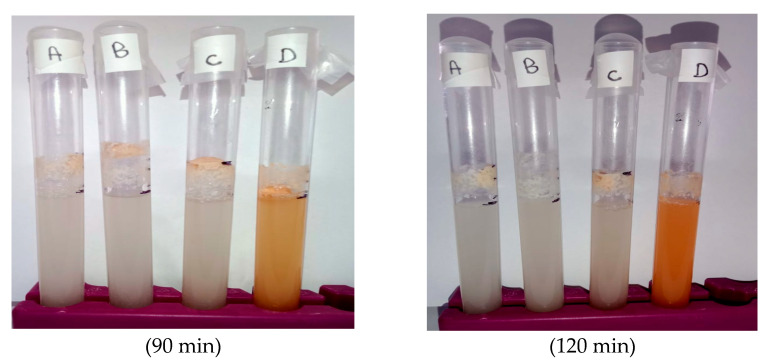
Macroscopic appearance of yeast fermentation tubes under different selenium nanoparticle (SeNP) treatments over time. A: control (0 mg·L^−1^); B: 0.5 mg·L^−1^ red SeNPs; C: 5 mg·L^−1^ red SeNPs; D: 50 mg·L^−1^ red SeNPs.

Immediately after mixing the required SeNP stock volume with the yeast growth medium, the tubes were covered and placed in an incubator set at 35 °C. Yeast growth and metabolic activity were monitored by visually assessing the qualitative indicator [9]: foam height at the following intervals: 0, 15, 30, 60, 90, and 120 min (Figure 1).

#### Optical Density Measurements

Yeast inoculums prepared in triplicate tubes: control—0 mg L^−1^; 0.5 mg L^−1^ red SeNPs; 0.5 mg L^−1^ grey SeNPs—were incubated at 35 °C with continuous shaking (40 rpm) for a total duration of 180 min; then, the growth was monitored at 30 min intervals. Before each measurement, cultures were gently mixed, and a 0.5 mL aliquot was withdrawn and diluted into 3.0 mL sterile water (final volume 3.5 mL; dilution factor DF = 7). Turbidity was measured at 600 nm using a PerkinElmer Lambda 2S UV/VIS spectrophotometer (PerkinElmer, Springfield, IL, USA) blanked with the corresponding uninoculated medium. The true OD_600_ was calculated by multiplying the measured value by the dilution factor. The maximum specific growth rate (µmax) was determined from the exponential growth phase. True OD_600_ values were transformed to their natural logarithm, and µmax was obtained as the slope of the linear region of the Ln(OD_600_) versus time curve, corresponding to the 0–30 min interval for all treatments. The specific growth rate was calculated using

$$\mu_{\max}=\frac{Ln({\mathrm{OD}}_{t2})-Ln({\mathrm{OD}}_{t1})}{t_{2}-t_{1}}$$

and is reported in units of h^−1^. All measurements were performed in triplicate, and results are expressed as mean ± standard deviation (SD).

### 2.4. Statistical Analysis

All statistical tests were conducted using GraphPad Prism version 9.5.0 (730), and the results are reported as mean values ± standard deviation of the mean. The differences between the groups were analyzed by a one-way analysis of variance (ANOVA), followed by pairwise comparisons tests, where statistical significance was measured using α < 0.05 and *p* value < 0.0001.

## 3. Results

### 3.1. Yeast Growth Dynamics Under Selenium Nanoparticle Treatments

**Figure 2:**
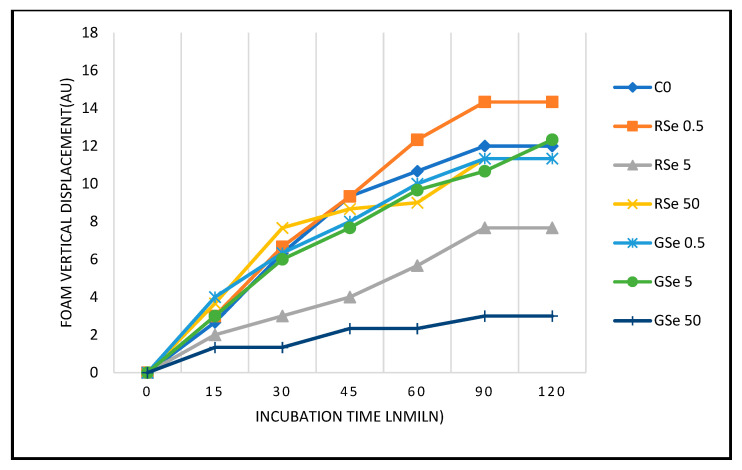
Growth curves of yeast during 120 min incubation. C0: control (0 mg·L^−1^); RSe 0.5: 0.5 mg·L^−1^ red SeNPs; RSe 5: 5 mg·L^−1^ red SeNPs; RSe 50: 50 mg·L^−1^ red SeNPs; GSe 0.5: 0.5 mg·L^−1^ grey SeNPs; GSe 5: 5 mg·L^−1^ grey SeNPs; GSe50: 50 mg·L^−1^ grey SeNPs.

Figure 2 shows the time-dependent yeast growth expressed as foam expansion distance under different selenium nanoparticle (SeNP) treatments. All treatments showed a progressive increase in foam distance over time, with clear variations among groups. The control and low-dose red SeNPs (RSe 0.5) showed the most rapid and extensive foam development, while the high-dose grey SeNPs (GSe 50) exhibited the slowest and smallest expansion throughout the 120 min incubation period. Control: Growth increased steadily, plateauing at ~12 AU by 90 min. RSe (0.5 mg·L^−1^) promoted yeast growth above control, reaching 14.3 AU at 90–120 min. RSe (5 mg·L^−1^) had a strong inhibitory effect, and growth plateaued at 7.7 AU. RSe (50 mg·L^−1^) caused slight stimulation initially (up to 7.7 AU at 30 min), but final growth (11.3 AU) remained close to the control. This distinct U-shaped trend suggests differential biological interactions across the tested concentration range. GSe (0.5 mg·L^−1^) enhanced early growth (highest at 15–30 min), plateauing at ~11.3 AU, comparable to control. GSe (5 mg·L^−1^) supported growth similarly to the control, reaching 12.3 AU at 120 min. GSe (50 mg·L^−1^) exhibited strong inhibition throughout, and growth did not exceed 3 AU.

### 3.2. Foam Distance After 120 Minutes and Growth Rate Estimation

**Figure 3:**
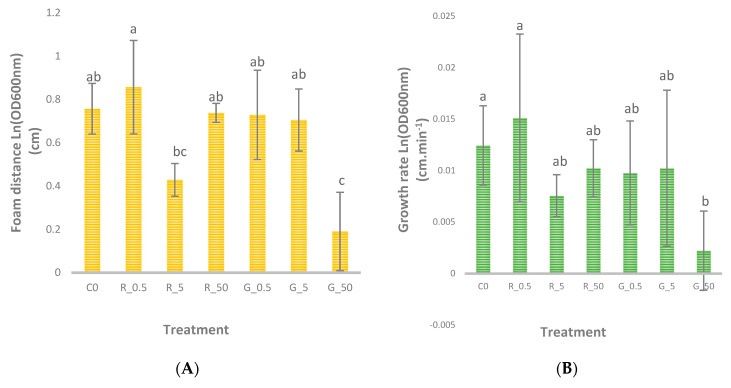
Growth response of yeast under different selenium nanoparticle (SeNP) treatments. (**A**) Foam distance formed after 120 min incubation under different SeNP treatments. (**B**) Growth rate of yeast (cm·min^−1^), as affected by red and grey SeNPs. C0: control (0 mg·L ^−1^); RSe 0.5: (0.5 mg·L ^−1^) red SeNPs; RSe 5: (5 mg·L ^−1^) red SeNPs; RSe 50: (50 mg·L ^−1^) red SeNPs; GSe 0.5: (0.5 mg·L ^−1^) grey SeNPs; GSe 5: (5 mg·L ^−1^) grey SeNPs; GSe50: (50 mg·L ^−1^) grey SeNPs. Different letters indicate significant differences (*p* < 0.05).

As shown in Figure 3A, foam distance after 120 min differed significantly (*p* < 0.05) among treatments. The largest foam distances were recorded in the control and RSe 0.5 groups, followed by RSe 50, GSe 0.5, and GSe 5, while the lowest expansion occurred in GSe 50. Treatments sharing different Tukey letters (a, b, c) indicate statistically distinct means. The observed pattern confirms that the SeNP form was the dominant factor affecting yeast performance. Red SeNPs promoted higher foam expansion, suggesting improved fermentation efficiency, whereas increasing concentrations of grey SeNPs reduced yeast activity. These differences can be explained by the higher colloidal stability and reactivity of amorphous red SeNPs compared with crystalline grey SeNPs.

The calculated growth rates (cm·min^−1^) are presented in Figure 3B, where significant differences (*p* < 0.05) were also detected among treatments. The highest growth rate occurred in the RSe 0.5 group, followed by the control, RSe 5, RSe 50, and grey SeNPs at moderate doses. The lowest rate was observed in the GSe 50 group, confirming the inhibitory effect of high-dose grey selenium. This pattern demonstrates that red SeNPs enhanced yeast proliferation and metabolic activity, particularly at low doses, while grey SeNPs at higher levels impaired growth. These results are consistent with the physicochemical behaviour of both forms—red SeNPs are typically smaller, more uniform, and possess higher surface energy, enhancing their biological interaction potential [10]. In contrast, the crystalline grey form tends to aggregate, lowering cellular uptake and reducing its stimulatory capacity [11].

### 3.3. Role of Foam Formation and SeNP Form

The foam height (or distance) directly reflects the rate of CO_2_ production during yeast fermentation. The greater foam expansion observed under low-level red selenium nanoparticles (SeNPs) indicates enhanced metabolic and fermentative activity, whereas the reduced foam formation at high grey SeNPs concentrations suggests metabolic inhibition or slower gas production, possibly due to oxidative stress or impaired cell wall permeability [5]. These findings highlight that the morphological form of selenium nanoparticles (red vs. grey) exerted a stronger influence on yeast growth than concentration alone. Red SeNPs appeared to stimulate yeast activity, likely due to their higher bioavailability and reactive surface characteristics, while grey SeNPs, particularly at higher concentrations, suppressed growth—potentially as a result of dose increase or oxidative stress limiting CO_2_ release and overall metabolic performance [12].

### 3.4. Growth Kinetics and Maximum Specific Growth Rate (µmax)

**Figure 4:**
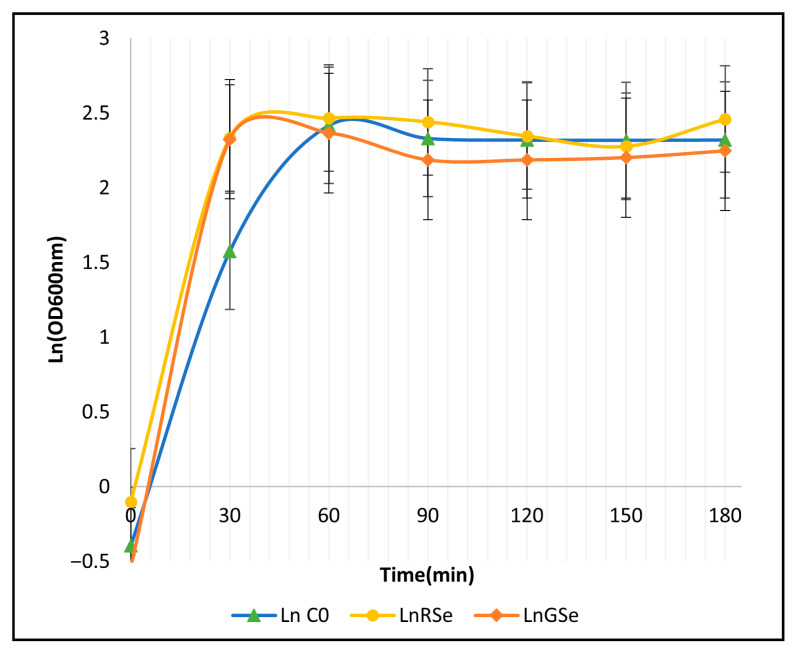
Growth curves of *Saccharomyces cerevisiae* under different selenium nanoparticle treatments. The natural logarithm of the optical density (OD600 nm) is plotted against time: Ln C0: control (0 mg·L^−1^); Ln RSe: 0.5 mg·L^−1^ red SeNPs; Ln GSe: 0.5 mg·L^−1^ grey SeNPs. Error bars represent the standard deviation (SD) of three replicates.

The growth curves expressed as Ln(OD_600_) revealed clear differences in the early exponential phase among the control, red SeNP, and grey SeNP treatments (Figure 4). All cultures entered exponential growth immediately after inoculation, but the steepness of the 0–30 min interval differed markedly between treatments. Grey SeNPs (0.5 mg L^−1^) showed the most rapid increase in Ln(OD_600_), followed by red SeNPs, whereas the control displayed the slowest early growth. After 30 min, the curves began to decelerate and gradually approached a plateau, indicating the transition to the stationary phase. Between 60 and 180 min, the treatments remained relatively stable, with red SeNPs maintaining slightly higher Ln(OD_600_) values than both grey SeNPs and the control, although the differences during the stationary phase were modest and partially overlapped with the variability among replicates.

The maximum specific growth rate (µmax) was calculated from the 0–30 min interval, which represented the linear portion of the exponential phase for all treatments. The grey SeNP treatment exhibited the highest µmax value (5.73 h^−1^), indicating the strongest stimulation of early yeast growth. Red SeNPs produced an intermediate growth rate (4.867 h^−1^), while the control showed the lowest µmax (3.939 h^−1^) (Table 1). These results demonstrate that both selenium nanoparticle forms enhanced early metabolic activity at the low concentration tested (0.5 mg L^−1^), with grey SeNPs exerting the greatest effect on exponential growth.

## 4. Discussion

The experimental evaluation of red and grey SeNPs revealed a clear distinction in their biological effects on S. cerevisiae, primarily governed by nanoparticle allotrope and concentration. The patterns observed in final foam distance and growth rate confirm that the SeNP allotrope was the dominant factor influencing yeast performance. The most striking result was the concentration-dependent toxicity exhibited specifically by grey SeNPs. At the highest dose (GSe 50), grey SeNPs caused a statistically significant reduction in growth rate, demonstrating their strong cytotoxic potential at elevated concentrations. In contrast, the lower and medium grey doses (GSe 0.5 and GSe 5) produced growth rates statistically similar to the control, suggesting that the toxic mechanism becomes evident only beyond a certain concentration threshold.

In sharp contrast, red SeNPs showed superior biocompatibility across the tested range. The dose–response profile exhibited a distinct U-shaped, non-monotonic pattern [13,14]: the low (0.5 mg·L^−1^) and high (50 mg·L^−1^) doses supported higher yeast growth, whereas the intermediate dose (5 mg·L^−1^) resulted in a modest reduction in activity. This slight dip at the medium dose may reflect a transitional metabolic stress response, consistent with mild hormesis. The recovery observed at the highest dose from amorphous selenium is likely due to increased nanoparticle aggregation, which reduces the effective bioavailable surface area and consequently diminishes toxicity. By comparison, grey SeNPs followed a consistent, monotonic inhibitory trend, with increasing concentrations producing progressively stronger suppression and maximal inhibition at 50 mg·L^−1^. This pattern suggests that grey SeNPs are more structurally stable and less prone to aggregation, enabling their toxic surface interactions—or the release of dissolved selenium species—to scale cumulatively with concentration [11]. Previous studies have shown that SeNPs can disrupt microbial membranes and DNA, generating reactive oxygen species (ROS) that induce oxidative stress, inhibit protein synthesis, and suppress microbial proliferation [12]. The shape and morphology of nanoparticles significantly affect their penetration, bioavailability, and antimicrobial activity [15,16,17]. Similarly, surface interactions play a critical role in SeNP toxicity [18], while nanoparticle surface charge and aggregation state influence membrane stability and cellular uptake [11]. Furthermore, selenium speciation affects its biological uptake and utilization even at adequate doses [19]. The crystalline status also determines nanoparticle reactivity—amorphous (red) and trigonal (grey) SeNPs tend to induce oxidative stress and show antimicrobial properties, whereas monoclinic SeNPs are less biologically active [20,21]. Microorganisms often transform red SeNPs into grey trigonal forms to reduce bioactivity, while yeast and plants can convert selenium into selenoamino acids, thus mitigating its toxicity [5]. Grey SeNPs produced the highest µmax during the first 30 min, whereas red SeNPs showed the highest growth and foam formation at 120 min. µmax reflects the short-term exponential growth rate, where grey SeNPs exerted a strong stimulatory effect. In contrast, the foam height and OD measurements at 120 min reflect the stationary phase, where red SeNPs supported higher metabolic activity and biomass maintenance. Thus, grey SeNPs enhance early growth kinetics, while red SeNPs promote sustained fermentation and long-term growth performance. Growth modulation in *S. cerevisiae* under exposure to selenium-based nanoparticles, including selenium sulphide forms or even inorganic forms, has been reported in earlier studies [22,23]. Overall, these results demonstrate that the morphological form of SeNPs plays a central role in modulating yeast growth and fermentation performance. While both forms exhibited concentration-dependent effects, red SeNPs supported fermentative activity, whereas grey SeNPs at high levels inhibited it.

## 5. Conclusions

This study successfully demonstrated that the allotropic state of selenium nanoparticles (SeNPs) is a critical determinant of their biological effect on *Saccharomyces cerevisiae* growth. Grey SeNPs exhibited concentration-dependent toxicity, with the highest dose causing a statistically significant growth reduction (GSe 50 group), confirming its cytotoxic potential. In sharp contrast, red SeNPs showed superior biocompatibility. The lowest red dose yielded the highest overall growth speed (RSe 0.5 group), suggesting a beneficial effect, while none of the red concentrations caused severe inhibition. Kinetic analysis showed that grey SeNPs accelerated early exponential growth, whereas red SeNPs supported higher metabolic activity and biomass stability at later stages. In summary, amorphous red SeNPs are less inhibitory and potentially more beneficial than their crystalline grey counterparts. These findings underscore the effect of both allotropic states of SeNPs. Future research should focus on elucidating the underlying cellular uptake and oxidative stress pathways responsible for these divergent toxicological profiles.

## Figures and Tables

**Table 1:** Maximum specific growth rate of *Saccharomyces cerevisiae* under different selenium nanoparticle treatments.

Group	Maximum Specific Growth Rate (μmax) (h^−1^)
Control (C0)	3.939
Red SeNPs (RSe 0.5)	4.867
Grey SeNPs (GSe 0.5)	5.73

## Data Availability

The original contributions presented in the study are included in the article, and further inquiries can be directed to the corresponding author.
